# Nurse-led weaning protocols—a systematic review and meta-analysis

**DOI:** 10.3389/fmed.2025.1514287

**Published:** 2025-08-01

**Authors:** Yingying Wang, Yan Wang, Su Gu, Liqun Zhu, Ruixiao Jia, Min Tan, Shaoyong Ma

**Affiliations:** ^1^Emergency Intensive Care Unit, Yijishan Hospital of Wannan Medical College, Wuhu, China; ^2^Nursing Department, Affiliated Hospital of Jiangsu University, Zhenjiang, China; ^3^Yancheng Clinical Medical College of Jiangsu University, Yancheng, China; ^4^Gastrointestinal Surgery, Zhejiang Cancer Hospital, Hangzhou, China; ^5^Department of Nursing, Affiliated Hospital of North Sichuan Medical College, Nanchong, China; ^6^School of Nursing, Wannan Medical College, Wuhu, China

**Keywords:** critical care nursing, systematic review, ventilator weaning, weaning protocol, mechanical ventilation

## Abstract

**Background:**

Research has demonstrated that protocol-directed weaning shortens the duration of mechanical ventilation (MV). Nurse-led weaning protocols have also been suggested to reduce MV duration in patients. Nevertheless, their implementation in mechanically ventilated patients is not widespread, and their clinical effectiveness remains uncertain.

**Aim:**

This study aimed to evaluate the effectiveness of nurse-led weaning protocols compared to physician-led weaning in mechanically ventilated patients.

**Methods:**

Eleven electronic databases (PubMed, Embase, Cochrane Library, CINAHL, EBSCO, Scopus, Web of Science, Sino Med, CNKI, VIP, and Wanfang) were systematically searched from their inception to January 20, 2024. Two researchers conducted a literature search and data extraction independently. We performed all statistical analyses using RevMan 5.1. The certainty of evidence was assessed using the Grades of Recommendation, Assessment, Development, and Evaluation (GRADE) profiler Guideline Development Tool.

**Results:**

Our analysis included a total of six relevant studies. The analysis of pooled data revealed that nurse-led weaning protocols were associated with a significant reduction in the duration of MV (MD = −1.78 days, 95% confidence interval (CI) = −3.08 to −0.48, I^2^ = 82%, and *p* = 0.007; five studies; moderate quality), length of stay (LOS) in the intensive care unit (ICU) (MD = −2.04 days, 95% CI = −2.65 to −1.44, I^2^ = 0%, and *p* < 0.00001; five studies; moderate quality), and LOS (MD = −2.54 days, 95% CI = −3.95 to −1.14, I^2^ = 0%, and *p* = 0.0004; three studies; low quality). Furthermore, nurse-led weaning protocols were associated with a reduced incidence of ventilator-associated pneumonia (VAP) (OR = 0.54, 95% CI = 0.31 to 0.96, I^2^ = 0%, and *p* = 0.03; 2 studies; moderate quality).

**Conclusion:**

Current evidence indicates that nurse-led weaning protocols have the potential to decrease the duration of MV, ICU LOS, LOS, and incidence of VAP in mechanically ventilated patients. However, these findings should be interpreted with caution due to the moderate-to-low quality of evidence and the limited number of available studies. Further high-quality, large-scale research is needed to confirm the effectiveness and safety of nurse-led weaning protocols in diverse clinical settings.

**Systematic review registration:**

Identifier: CRD42023487455, https://www.crd.york.ac.uk/prospero.

## 1 Introduction

Mechanical ventilation (MV) is the primary treatment for critically ill patients in the intensive care unit (ICU) (1). Healthcare professionals have concentrated on identifying the optimal timing for weaning patients from MV (2–5). Cederwall et al. (6) have demonstrated that the duration of weaning constitutes more than 50% of the total MV time. Prolonged MV is linked to extended lengths of stay (LOS), increased medical costs, and various complications, including ventilator-associated pneumonia (VAP) (7). Therefore, timely weaning is essential when the patient’s underlying condition improves or stabilizes, facilitating the transition from full respiratory support to spontaneous breathing (8, 9). A multicenter survey indicates that approximately 25% of ICU patients encounter weaning difficulties or delays, leading to extended MV, prolonged ICU stays, and increased mortality rates (10).

Several studies have demonstrated that protocolized weaning strategies can effectively reduce complications, such as VAP, shorten the duration of MV, and decrease the rate of reintubation (11–13). Significant variations in weaning strategies arise due to differences in clinicians’ knowledge, skills, and attitudes. Foster et al. (14) pioneered the development of a weaning protocol, which is a structured guideline for reducing or discontinuing MV support. This protocol involves three steps: evaluating the fulfillment of weaning criteria, reducing ventilatory support, and removing the endotracheal tube (15). Protocolized weaning minimizes the impact of subjective judgment and experience on weaning procedures and empowers nurses and respiratory therapists to actively participate in the weaning process.

Several studies have demonstrated that nurse-led weaning can safely shorten the duration of MV compared to physician-led weaning (16–18). These findings were corroborated by a systematic review conducted in 2019 (19). However, a recent retrospective cohort study reported that nurse-led weaning did not significantly reduce the duration of MV when compared to physician-led weaning (20). In light of the ongoing debate regarding the effectiveness of nurse-led weaning in mechanically ventilated patients, and considering the limitations of previous systematic reviews—which have mainly focused on adult MV patients and included a limited number of studies with low-quality evidence (19)—there is an urgent need to update the evidence in this field.

This study aimed to systematically review the empirical literature to assess the effectiveness of nurse-led weaning in mechanically ventilated patients and to generate high-quality evidence for clinical practice through a meta-analysis.

## 2 Methods

This systematic review and meta-analysis were registered on the International Prospective Register of Systematic Reviews (PROSPERO number: CRD42023487455) and were conducted and reported according to the Preferred Reporting Items for Systematic Reviews and Meta-Analysis (PRISMA) guidelines (21).

### 2.1 Search strategy and selection criteria

The search strategy involved querying seven English databases—PubMed, Embase, Cochrane Central Register of Controlled Trials, CINAHL, EBSCO, Scopus, and Web of Science—and four Chinese databases—Chinese Biomedical Literature Database, Wanfang Data, Chinese National Knowledge Infrastructure, and Chinese Science and Technology Periodical Database—as well as grey literature sources, including Open Grey^1^ and Grey Literature Report,^2^ from the inception of each database to January 20, 2024. Search terms included a combination of medical subject headings and keywords related to weaning from mechanical ventilation, such as “Ventilator Weaning,” “Mechanical,” “Artificial,” “Nurses,” “Nurse-led,” “Nurse-led care,” “Protocol,” “Medical,” “Physician,” and “Doctor-led care.” Additionally, manual searches were conducted based on the reference lists of eligible studies that were not identified through the initial search strategy. Two authors (YW and YYW) independently evaluated titles, abstracts, and full texts for eligibility, with a third researcher (LZ) consulted in cases of disagreement or uncertainty.

All retrieved studies were imported into EndNote 20 for management and checked for duplicates. Titles and abstracts were independently screened by two reviewers (YW and YYW). Similarly, full-text articles were independently screened by the same reviewers. In cases of discrepancies, consensus meetings were held between the paired reviewers. If consensus could not be reached, a third reviewer (LZ) was consulted to make the final decision on article inclusion.

The study included peer-reviewed research meeting the following inclusion criteria: (1) randomized controlled trials (RCTs) and cohort studies that focused on patients requiring MV in the ICU, (2) protocolized weaning led by nurses with a comparison to physician-led weaning, and (3) reporting at least one of the following outcomes: duration of MV, duration of weaning, ICU and hospital LOS, ICU and hospital mortality rates, incidence of VAP, and reintubation rate. The peer-reviewed research studies included in this review did not set specific age limitations for patients. Studies focusing on patients requiring permanent MV, such as those receiving home ventilation, were excluded. In all the peer-reviewed research papers included in this study, the weaning protocols were specifically and directly adjusted by nurses or doctors. Studies using ventilators with quasi-automatic or ventilator-assisted weaning modes were excluded.

### 2.2 Data extraction and quality assessment

Two reviewers (YW and YYW) independently extracted data from the included studies using a data extraction sheet specifically designed for this systematic review. Extracted information encompassed publication details (author, study year, and country), study design, study setting, population (sample size), intervention characteristics (methods and duration), control group details (methods, duration, and frequency), main outcomes, and assessments. In cases of disagreement regarding extracted information, consensus was reached through discussion with a third reviewer (LZ). If necessary, authors were contacted via email for additional data. When studies reported multiple intervention groups, the group involving nurse-led weaning interventions was selected.

Two independent reviewers (YW and YYW) conducted the quality assessment of the included articles. A third reviewer (LZ) was consulted to resolve inconsistencies between the two reviewers. The risk of bias in randomized controlled trials (RCTs) was assessed using the Cochrane Risk-of-Bias Tool version 2 (ROB 2) (22), while the risk of bias in cohort studies was evaluated using the Newcastle–Ottawa Scale (NOS) (23). The NOS includes eight entries that cover three aspects: the selection of study subjects in the exposed and non-exposed groups, the comparability between these groups, and the assessment of exposure outcomes.

The evidence for each outcome was assessed using the Grading of Recommendations Assessment, Development, and Evaluation (GRADE) profiler Guideline Development Tool, which categorizes evidence into four levels: very low, low, moderate, and high.

### 2.3 Statistical analysis

Meta-analysis was conducted for each outcome using Review Manager 5.1 software. Continuous variables, including duration of MV, time off the machine, ICU LOS, and hospital LOS, were reported as mean differences (MD), medians, and interquartile ranges (IQRs) with a 95% confidence interval (CI) through the application of the inverse variance method. Cochrane’s Q test and the I^2^ statistic were used to assess the proportion of variance in observed effects attributed to true effects. A *p*-value of less than 0.10 or an I^2^ value of 50% or higher was considered indicative of substantial heterogeneity (24). A random-effects model was adopted if *p* < 0.10 or I^2^ ≥ 50%; otherwise, a fixed-effects model was used. For binary outcomes, such as the incidence of ventilator-associated pneumonia and mortality, effect sizes were calculated using risk ratios (RR) or odds ratios (OR) based on the number of events and sample sizes across different groups. Meta-analysis was performed when at least two studies reported the same outcome. Outcomes not suitable for meta-analysis were presented narratively. A *p*-value of less than 0.05 was deemed statistically significant.

## 3 Results

### 3.1 Search strategy

**Figure 1:**
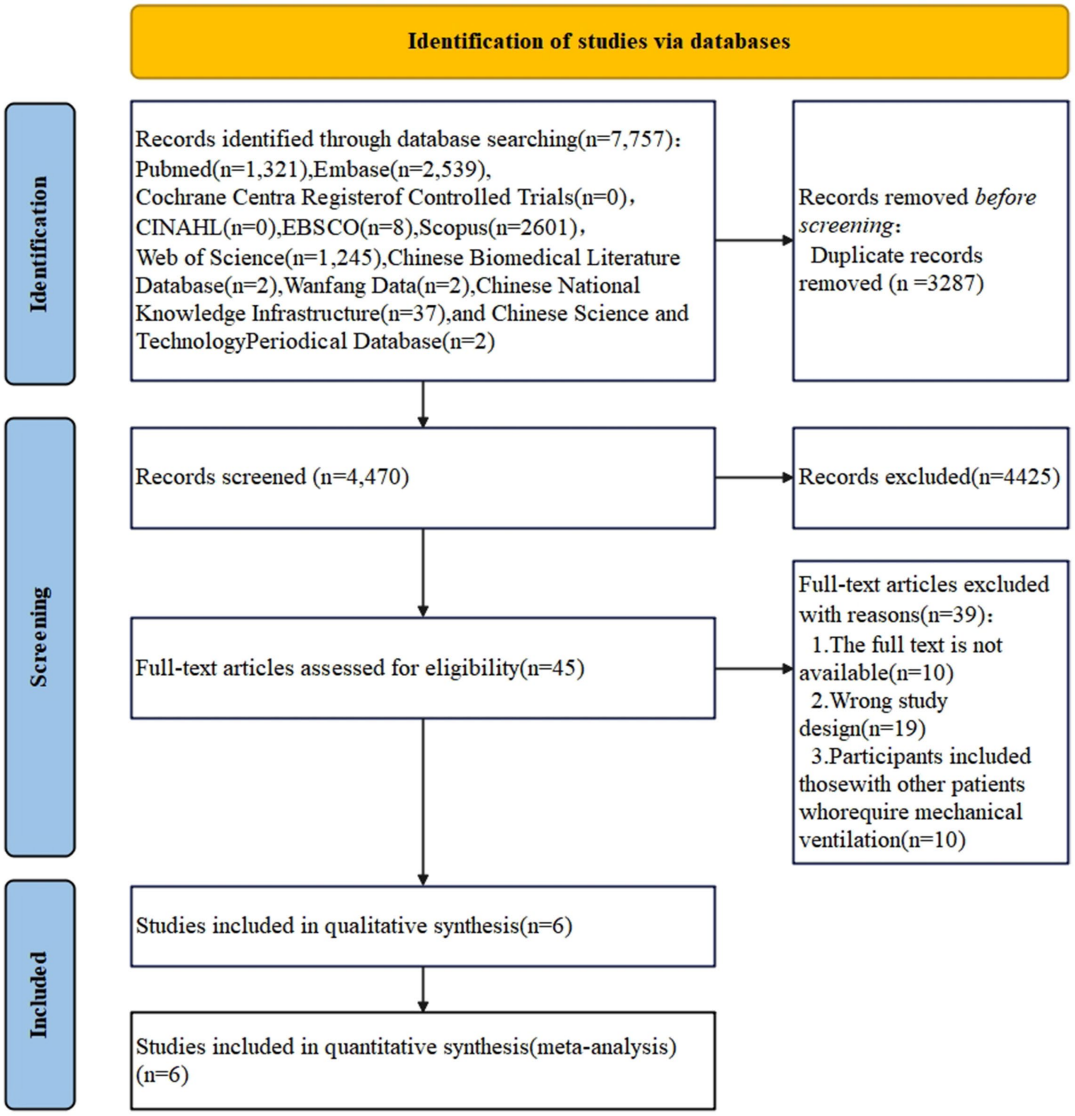
PRISMA diagram.

The initial online search yielded a total of 7,757 records. After removing duplicates, the titles and abstracts of 4,470 papers were screened for potential inclusion, with 4,425 articles deemed irrelevant for this review. The remaining 45 articles were evaluated in full text. Additionally, no relevant articles were identified using the ‘snowball’ technique. Ultimately, six studies were included in the systematic review and meta-analysis (see Figure 1).

### 3.2 General study characteristics

The six studies were conducted in South Korea, Thailand, the United Kingdom (UK), France, the United States of America (USA), and the Netherlands (16–18, 25). Of these, three studies were randomized controlled trials, while the remaining three were cohort studies.

#### 3.2.1 Characteristics of participants

A total of 1,063 mechanically ventilated patients were included across the six studies, with sample sizes ranging from 7 to 424. The age of the mechanically ventilated patients ranged from 1.00 to 82.00 years, with a mean age of 61.04 ± 15.2 years. Of these patients, 41.67% were female. Four of the six studies were conducted in the ICU (16–18, 25), while the remaining two were conducted in the pediatric intensive care unit (PICU).

#### 3.2.2 Characteristics of interventions

Various withdrawal strategies were employed across the studies, with four studies utilizing spontaneous breathing trials (SBT) (16–18, 25). Danckers et al. (17) and Roh et al. (18) implemented nurse-led withdrawal protocols, which included continuous positive airway pressure-SBT. For patients meeting withdrawal eligibility criteria, SBT lasted for 30–120 min through a ventilator circuit. In the control group, the initiation of weaning is primarily determined by the attending physician’s clinical judgment. Physicians typically use pressure support ventilation (5–8 cm H_2_O) during the weaning process, and continuous positive airway pressure may be applied at their discretion. Nurses are generally not involved in assessing readiness for weaning, initiating the process, or managing its progression. Tonnelier et al. (25) conducted SBT using a T-piece, deeming the trial successful if it lasted 90 min without clinical intolerance. In the control group, the weaning mode (volume-assisted controlled ventilation, pressure support ventilation, or T-piece) was selected based on the clinician’s discretion. Chaiwat et al. (16) screened mechanically ventilated patients for SBT daily. If any component of the daily screening was unmet, the patient did not undergo SBT that day and continued screening the next morning. Conversely, if the ventilator support pressure reached 7 cm H_2_O and the positive end-expiratory pressure reached 5 cm H_2_O for 120 min without clinical intolerance, the SBT was considered successful. In the control group, the initiation of weaning was guided by the clinical judgment of the attending physician. A standardized weaning protocol was not specified in the study. Duyndam et al. (20) employed a two-step approach to withdrawal: first, gradually ceasing oxygen and positive end-expiratory pressure; then selecting volume support under pressure-regulated volume control or pressure support under pressure control, gradually reducing pressure or volume. In the control group, the decision to initiate weaning was based on the clinical judgment of the attending physician. The specific weaning method was not explicitly detailed in the manuscript. Rushforth (26) assessed patients every 4 h, adjusting ventilator-related parameters based on blood gas analysis results. When patients met withdrawal criteria, blood gas analysis was rechecked 30 min later to confirm withdrawal appropriateness. In the control group, the weaning process was guided by the physician’s clinical experience, without the application of a standardized protocol.

#### 3.2.3 Characteristics of outcomes

The outcomes included duration of MV, ICU LOS, hospital LOS, weaning time, incidence of VAP, reintubation rate, ICU mortality rate, and hospital mortality rate. Detailed information is available in Table 1.

**Table 1:** Characteristics of the included studies.

Study (author, year, country)	Study design	Sample size (*n*)	Participants	Interventions	Outcome measures
Roh et al. (18), Korea	RCT	122	Adult patients on mechanical ventilation for more than 12 h or less than 14 days (short-and long-term period)	I: *n* = 61 critical care nurse-driven ventilator-weaning protocol	①②③④⑧
				C: *n* = 61 Physician delivered Protocol	
Chaiwat et al. (16), Thailand	RCT	100	Intra-abdominal surgical adult patients requiring mechanical ventilation for more than 24 h	I: *n* = 51 protocol-based nurse-directed weaning	①⑥
				C: *n* = 49 physician-directed weaning	
Rushforth et al. (26), UK	RCT	7	Infants under 1 year of age, less than 10 kg in weight, and admitted to the PICU at LGI with a primary respiratory problem	I: *n* = 3 nurse-led (protocol-directed)	②③④
				C: *n* = 4 medical-led weaning	
Duyndam et al. (20), Netherland	Cohort study	424	Children up to the age of 18 years receiving iMV	I: *n* = 212 nurse-driven weaning protocol	①②⑥
				C: *n* = 212 usual physician-driven weaning protocol	
Tonnelier et al. (25), France	Cohort study	208	Adult patients were mechanically ventilated through an endotracheal tube and required MV for longer than 48 h.	I: *n* = 104 nurse-driven ventilator-weaning protocol	①②⑤⑥⑦
				C: *n* = 104 conventional physician-directed weaning (historical control)	
Danckers et al. (17), USA	Cohort study	202	Mechanically ventilated adult patients for more than 24 h (short-term period)	I: *n* = 102 nurse-driven ventilator-weaning protocol	①②③⑤⑥⑧
				C: *n* = 100 Physician-driven ventilator weaning (historical control)	

RCT, randomized controlled trial.

I, Intervention group; C, Control group; ①: Duration of MV; ② ICU LOS; ③ hospital LOS; ④ Weaning time; ⑤ VAP; ⑥ Reintubation rate; ⑦ ICU Mortality; ⑧ Hospital mortality.iMV, invasive mechanical ventilation.

### 3.3 Risk of bias and quality of the evidence

The results of the risk of bias assessment are presented in Tables 2, 3. All three randomized controlled trials were identified as having a high risk of bias (16, 18, 26). Regarding the randomization process, one study raised concerns because it reported only the baseline characteristics of participants in the final analysis (26). The risk of bias concerning intended interventions (effect of assignment to intervention) in three studies was evaluated as having some concerns. This was due to the lack of mention of appropriate analysis to estimate the effect of assignment to intervention in two studies (18, 26) and insufficient information in one study (16). The outcome measurement in three studies was considered to have a high risk of bias, as the outcome assessor was also the study participant (16, 18, 26). Three studies presented some concerns regarding the bias in reported results due to the absence of a registered protocol, and the reported intent of the analysis was insufficiently detailed for evaluation (16, 18, 26).

**Table 2:** Methodological quality assessment results of randomized controlled trials (*n* = 3).

Study, year	Randomization process	Bias due to deviations from intended interventions (effect of assignment to intervention)	Bias due to deviations from intended interventions (effect of adhering to the intervention).	Bias due to missing data	Bias in the measurement of outcomes	Bias in the selection of the reported result
Rushforth et al. (26)	Some concerns	Some concerns	Low risk of bias	Low risk of bias	High risk of bias	Some concerns
Chaiwat et al. (16)	Low risk of bias	Some concerns	Low risk of bias	Low risk of bias	High risk of bias	Some concerns
Roh et al. (18)	Low risk of bias	Some concerns	Low risk of bias	Low risk of bias	High risk of bias	Some concerns

**Table 3:** Methodological quality evaluation results of cohort studies (*n* = 3).

Study, year	①	②	③	④	⑤	⑥	⑦	⑧	Score	Quality grade
Tonnelier et al. (25)	1	1	1	1	2	1	0	1	8	A
Danckers et al. (17)	1	1	1	1	2	1	0	1	8	A
Duyndam et al. (20)	1	1	1	1	2	1	0	1	8	A

① Representativeness of the exposed cohort; ② Selection of the non-exposed cohort; ③ Ascertainment of exposure; ④ Demonstration that the outcome of interest was not present at the start of the study; ⑤ Comparability of cohorts on the basis of the design or analysis; ⑥ Assessment of outcome; ⑦ Was follow-up long enough for outcomes to occur; ⑧ Adequacy of follow-up of cohort.

For the three cohort studies, the risk of bias was found to be low, and the overall quality was considered acceptable. The results of the risk of bias assessment for these studies are presented in Table 3. None of the three studies (17, 20, 25) mentioned whether follow-up would occur, resulting in a score of 0 for the item “Was follow-up long enough for outcomes to occur.” The grade of evidence ranged from very low to medium, as shown in Table 4.

**Table 4:** GRADE evidence profile of nurse-led withdrawal program vs. control.

Outcome measure		Duration of MV	Weaning time	ICU stay	Length of stay	Reintubation rate	VAP incidence	ICU mortality	Hospital mortality
Certainty assessment	Number of studies	5	2	5	3	4	2	1	2
	Study design	2 RCT 3 NRCT	2 RCT	2 RCT 3 NRCT	2 RCT 1 NRCT	1 RCT 3 NRCT	2 NRCT	1 NRCT	1 RCT 1 NRCT
	Risk of bias	Serious^a^	Serious^b^	Serious^a^	Serious^a^	Serious^c^	Serious^c^	Serious^c^	Serious^a^
	Inconsistency	Not serious	Serious^d^	Not serious	Not serious	Serious^d^	Not serious	Not serious	Serious^d^
	Indirectness	Not serious	Not serious	Not serious	Not serious	Not serious	Not serious	Not serious	Not serious
	Imprecision	Not serious	Serious^e^	Not serious	Serious^e^	Not serious	Not serious	Serious^e^	Serious^e^
	Publication bias	None	None	None	None	None	None	None	None
Number of patients	Experimental group	530	64	482	166	469	206	104	163
	Control group	526	65	481	165	465	204	104	161
Effect	Relative (95%CI)								
	Absolute (95% CI)	MD 1.78 lower (3.08 lower to 0.48 lower)	MD 11.04 lower (58.90 lower to 36.82 higher)	MD 2.04 lower (2.65 lower to 1.44 lower)	MD 2.54 lower (3.95 lower to 1.14 lower)	OR 1.15 (0.74 to 1.79)	OR 0.54 (0.31 to 0.96)	OR 1.43 (0.44 to 4.66)	OR 0.86 (0.48 to 1.55)
Certainty of evidence (GRADE)	⊕⊕⊕⊙	⊕⊙⊙⊙	⊕⊕⊕⊙	⊕⊕⊙⊙	⊕⊕⊙⊙	⊕⊕⊕⊙	⊕⊕⊙⊙	⊕⊙⊙⊙	
	Moderate	Very low	Moderate	Low	Low	Moderate	Low	Very low	
Importance	Critical	Critical	Critical	Critical	Critical	Critical	Critical	Critical	

RR, rate ratio; OR, odds ratio; RCTs, randomized controlled trials; NRCTs, non-randomized controlled trials; CI, confidence interval, and MD, mean difference.

⊕⊕⊕⊕ indicates high-quality evidence.

⊕⊕⊕⊙ indicates moderate-quality evidence.

⊕⊕⊙⊙ indicates low-quality evidence.

⊕⊙⊙⊙ indicates very low-quality evidence.

a Downgraded one level because of risk of bias: lack of blinding (caregivers are aware of the arm to which patients are allocated).

b Downgraded one level because of failure to adequately control confounding.

c Downgraded one level because of differences in measurement of exposure (recall bias in case control studies).

d Downgraded one level because of inconsistency in absolute effect.

e Downgraded one level because of wide confidence intervals and few events.

### 3.4 Outcomes

#### 3.4.1 Duration of MV (days)

**Figure 2:**
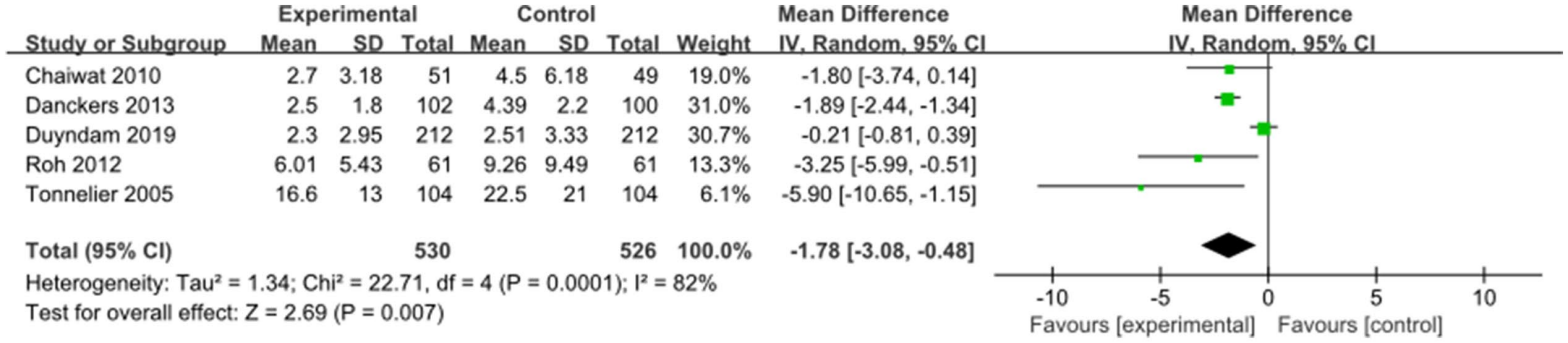
Forest plots—the effect of nurse-led weaning protocols on the duration of MV.

Five studies examined the effect of nurse-led weaning protocols on the duration of MV. Pooled results indicated that nurse-led weaning protocols could reduce the duration of MV (MD = −1.78, 95% CI: −3.08 to −0.48; *p* = 0.007, I^2^ = 82%; see Figure 2). Despite considerable heterogeneity, a meta-analysis was conducted due to the consistent direction of the effect. The overall certainty of this body of evidence was rated as moderate, primarily due to significant bias limitations.

#### 3.4.2 Weaning time (h)

**Figure 3:**
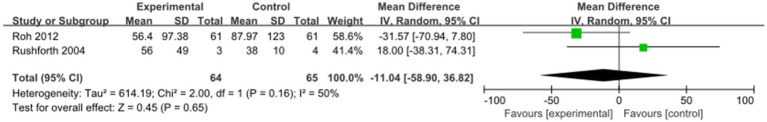
Forest plots—the effect of nurse-led weaning protocols on weaning time.

Two studies examined the impact of nurse-led weaning protocols on weaning time. The protocols did not significantly reduce weaning time (MD = −11.04, 95% CI: −58.90 to 36.82; *p* > 0.05, I^2^ = 50%; see Figure 3). Due to significant limitations related to bias, indirectness, and imprecision, the overall quality of this evidence was deemed very low.

#### 3.4.3 ICU LOS

**Figure 4:**
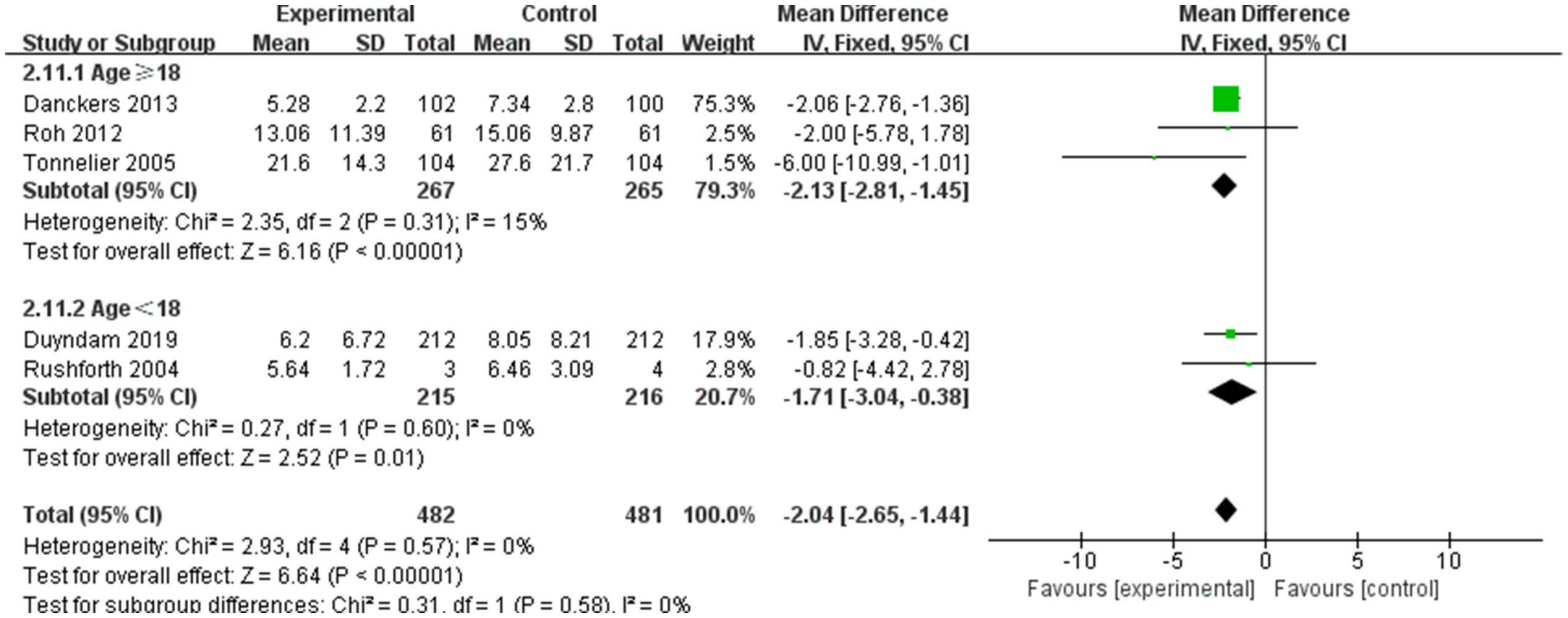
Forest plots—the effect of nurse-led weaning protocols on ICU LOS.

Five studies assessed the impact of nurse-led weaning protocols on ICU LOS. Pooled results indicated that nurse-led weaning protocols could reduce ICU LOS for mechanically ventilated patients (MD = −2.04, 95% CI: −2.65 to −1.44; *p* < 0.00001, I^2^ = 0%; see Figure 4).

Subgroup analysis for patients aged 18 years and older indicated that nurse-led weaning protocols could reduce ICU LOS for adult mechanically ventilated patients (MD = −2.13, 95% CI: −2.81 to −1.45; p < 0.00001, I^2^ = 0%; see Figure 4). The protocols also shortened ICU LOS for pediatric mechanically ventilated patients (MD = −1.71, 95% CI: −3.04 to −0.38; *p* = 0.01, I^2^ = 0%; see Figure 4). Due to significant limitations in bias, the overall certainty of this body of evidence was deemed moderate.

#### 3.4.4 Hospital LOS (days)

**Figure 5:**
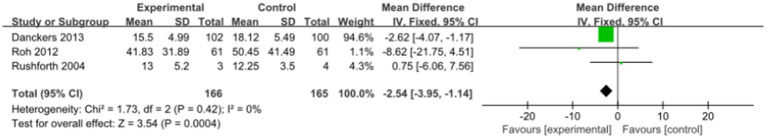
Forest plots—the effect of nurse-led weaning protocols on hospital LOS.

Three studies assessed the impact of nurse-led weaning protocols on hospital LOS. Pooled results indicated that nurse-led weaning protocols could reduce hospital LOS (MD = −2.54, 95% CI: −3.95 to −1.14; *p* = 0.0004, I^2^ = 0%; see Figure 5). Due to significant limitations related to bias and imprecision, the overall quality of this evidence was deemed low.

#### 3.4.5 Reintubation rate

**Figure 6:**
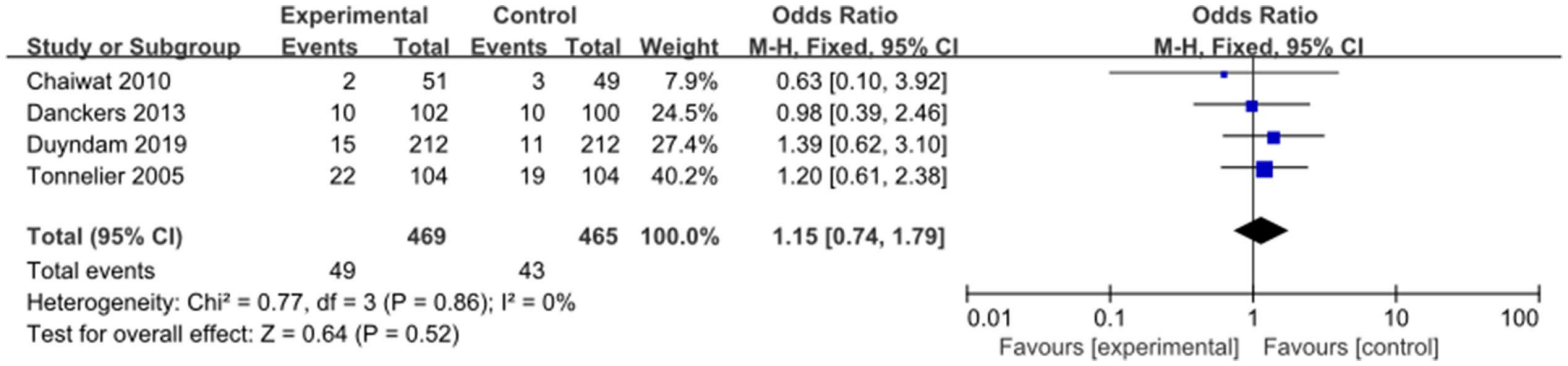
Forest plots—the effect of nurse-led weaning protocols on reintubation rate.

Four studies evaluated the impact of nurse-led weaning protocols on the reintubation rate. Pooled results indicated that nurse-led weaning protocols did not reduce the reintubation rate (OR = 1.15, 95% CI: 0.74 to 1.79; *p* = 0.52, I^2^ = 0%; see Figure 6). Due to significant limitations related to bias and inconsistency, the overall quality of this evidence was deemed low.

#### 3.4.6 VAP incidence

**Figure 7:**
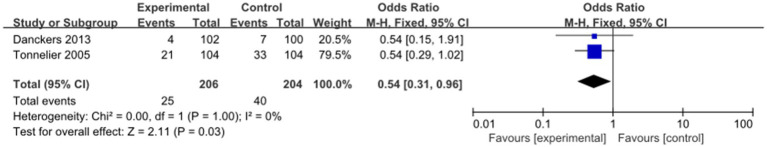
Forest plots—the effect of nurse-led weaning protocols on VAP incidence.

Two studies evaluated the impact of nurse-led weaning protocols on the incidence of VAP. Pooled results indicated that nurse-led weaning protocols reduced the incidence of VAP (OR = 0.54, 95% CI: 0.31 to 0.96; *p* = 0.03, I^2^ = 0%; see Figure 7). Due to significant limitations related to bias, the overall certainty of this body of evidence was deemed moderate.

#### 3.4.7 ICU mortality rate

One study evaluated the impact of nurse-led weaning protocols on ICU mortality rates (25). In this study, the ICU mortality rate was 7% in the nurse-led weaning group and 5% in the physician-led group, with no statistically significant difference observed (*p* = 0.92). Thus, nurse-led weaning protocols were found to have no significant effect on ICU mortality rates. However, the study had significant limitations, including risks of bias and imprecision, resulting in a low overall certainty of evidence.

#### 3.4.8 Hospital mortality rate

**Figure 8:**
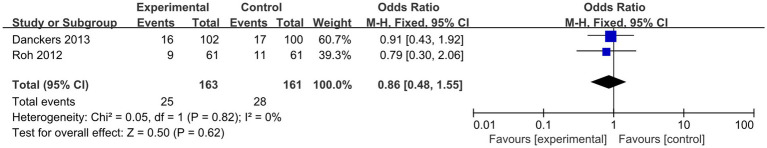
Forest plots—the effect of nurse-led weaning protocols on hospital mortality rate.

Two studies assessed the impact of nurse-led weaning protocols on hospital mortality rates. Pooled analysis results indicated that nurse-led weaning protocols had no significant effect on hospital mortality rates (OR = 0.86, 95% CI: 0.48 to 1.55; *p* = 0.62, I^2^ = 0%; see Figure 8). However, due to significant limitations related to bias, indirectness, and imprecision, the overall certainty of evidence was deemed very low.

## 4 Discussion

In this systematic review, we assessed evidence from three randomized controlled trials and three cohort studies regarding the impact of nurse-led weaning protocols on MV patients compared to physician-led weaning protocols. Our analysis suggests that a nurse-led weaning regimen may be associated with reductions in the duration of MV, ICU LOS, hospital LOS, and the incidence of VAP. However, these regimens did not show significant effects on weaning time, reintubation rates, ICU mortality, or hospital mortality. Nevertheless, these results should be interpreted with caution due to important limitations in the current evidence base. Specifically, the small number of included studies and their variable methodological quality limit the strength and generalizability of our conclusions. Moreover, the considerable heterogeneity in study design, clinical settings, and implementation fidelity across studies further complicates the interpretation of pooled outcomes. Therefore, further high-quality, large-scale randomized controlled trials are warranted to provide more robust evidence on the effectiveness and safety of nurse-led weaning protocols.

### 4.1 MV durations

Among the studies included in this review, five evaluated the duration of MV. Overall, four of these studies reported that nurse-led weaning protocols significantly reduced the duration of MV compared to physician-led weaning protocols (16–18, 25). Conversely, one study by Duyndam et al. (20) found no significant difference in MV duration between nurse-led and physician-led weaning protocols.

The observed variations in these findings can be attributed to several factors. Primarily, differences exist among nurses regarding their education level, attitude, autonomy, and collaboration. For instance, the study by Duyndam et al. (20) specifically revealed that, despite efforts by nurse managers and ventilation practitioners to educate and encourage nurses to use nurse-led weaning protocols, nurses still lacked confidence in implementing this approach. Second, the same study also indicated that many patients met the extubation criteria during nighttime hours. However, due to physicians’ cautious approach toward nighttime extubation—attributed to lower staffing ratios compared to daytime—the decision was often made to postpone extubation until the following day. Evidently, this delay in extubation may lead to a prolonged duration of MV. Finally, Duyndam et al. (20) further demonstrated that there was no significant difference in outcomes between patients extubated using supported mode and those extubated using low-pressure control mode. Moreover, this study suggested that transitioning from low-pressure control mode to supported mode during extubation might also contribute to an extended duration of MV.

Despite these nuances, our findings from the pooled data consistently support the use of nurse-led weaning protocols, aligning with earlier randomized controlled trials (27–29) and corroborated by non-randomized controlled trials (30, 31). Nevertheless, these findings should be interpreted with caution due to significant heterogeneity among the studies (I^2^ = 82%). Indeed, several potential sources contribute to this heterogeneity.

Initially, considerable variability was observed in patient populations among the included studies. For example, some investigations focused on adults, while others included pediatric patients; these groups typically differ in physiological characteristics, underlying comorbidities, and responses to weaning protocols. Furthermore, the intervention protocols themselves were not entirely uniform, as nurse-led weaning procedures varied in terms of protocol structure, nurse autonomy, criteria for weaning initiation, and interdisciplinary collaboration; such protocol differences are likely to influence weaning outcomes in multiple ways. In addition, differences in usual care among the comparator groups may have contributed to heterogeneity, since “standard care” could encompass diverse practices across various healthcare systems and institutions. Beyond these clinical factors, varying methodological quality among studies—including differences in study design, sample size, and risk of bias—may also have introduced further variability in the results.

Consistent with our findings on MV duration, a recent systematic review, encompassing 17 randomized controlled trials with 2,434 critically ill adult patients, compared protocolized weaning to non-protocolized weaning. The review demonstrated that weaning protocols reduced the duration of MV by an average of 26% compared to non-protocolized weaning (32).

Ultimately, the implementation of a nurse-led weaning protocol fundamentally contributes to a reduction in the duration of mechanical ventilation. The potential mechanisms underlying this outcome are discussed below. First, nurse-led protocols often involve more frequent and systematic patient assessments than standard physician-driven practices. Nurses at the bedside are able to promptly recognize early signs of patient readiness for weaning and initiate protocolized steps without unnecessary delays. This real-time responsiveness can shorten the pre-weaning phase and accelerate patient progression toward weaning initiation. Second, the implementation of structured weaning protocols empowers nurses to make autonomous decisions within predefined criteria, thereby reducing possible delays caused by hierarchical communication or awaiting physician orders. This efficiency throughout the mechanical ventilation process likely contributes to an overall reduction in ventilation time. Third, nurse-led protocols commonly emphasize interdisciplinary teamwork and communication, which can improve coordination of care (e.g., physiotherapy, nutrition, medication optimization). As a result, patients may reach ventilatory milestones more efficiently, further reducing unnecessary ventilator days not only during weaning but throughout the entire course of mechanical ventilation. Finally, these protocols may facilitate earlier recognition and management of non-respiratory barriers to weaning (such as delirium, sedation, or hemodynamic instability), as nurses routinely monitor and address such factors according to protocol prompts. Taken together, these multifaceted factors suggest that the benefit of nurse-led weaning protocols in reducing total MV duration extends not merely to the technical weaning phase but also to the overall care process before and during withdrawal from ventilation support.

### 4.2 Impact of nurse-led weaning protocols on weaning time

The meta-analysis indicated that nurse-led weaning protocols did not significantly reduce weaning time. Among the included studies, one reported a significant reduction in weaning time with nurse-led weaning compared to physician-led weaning (18), while another found no significant difference (26). Although the pooled data showed no significant difference in weaning time between nurse-led and physician-led weaning protocols, these findings should be interpreted with caution.

Several important considerations should be noted regarding the studies by Roh et al. (18) and Rushforth (26). First, the study by Roh et al. (18) focused on adult subjects, whereas the study by Rushforth (26) specifically examined infants. This difference in the age groups of subjects may influence the outcomes and conclusions. Second, variations existed in the intervention factors across the studies, including criteria for weaning readiness, ventilation modes, and parameters used. These differences in study protocols can introduce variability in results, making it challenging to draw definitive conclusions. Third, it is important to note that the study by Rushforth (26) had a limited sample size. Although the randomized controlled trial initially aimed to include 20 infants (10 in each group), only 8 infants were ultimately included. Such a small sample size can affect the statistical power and generalizability of the findings. Considering these factors—the differences in study subjects, variations in intervention factors, and the limited sample size—it is difficult to establish conclusive evidence regarding the effectiveness of nurse-led weaning protocols in reducing weaning time.

A study by Piotto et al. (33) demonstrated that protocolized weaning based on SBT significantly reduced weaning time in cardiac patients. Additionally, a systematic review by Blackwood et al. (32) revealed that protocolized weaning from mechanical ventilation (MV) reduced weaning time by an average of 70% compared to non-protocolized approaches. However, it is important to note significant heterogeneity among the effect estimates of the included studies (97%), indicating the need for caution when interpreting the findings.

### 4.3 Impact of nurse-led weaning protocols on ICU and hospital LOS

Building upon the observed reduction in MV duration, our meta-analysis further supports the efficacy of nurse-led weaning protocols in significantly reducing ICU LOS. Our findings align with those of Smyrnios et al. (34), who also reported a reduction in ICU LOS with nurse-led weaning protocols. However, a divergence from other studies is apparent; for instance, Ely et al. (27), Kollef et al. (28), Grap et al. (35), and Roh et al. (18) found no significant decrease in ICU LOS despite a reduction in MV duration. Furthermore, a notable finding of our study is the observed significant reduction in hospital LOS, which contradicts the findings of early research by Smyrnios et al. (34) and Kollef et al. (28).

### 4.4 Impact of nurse-led weaning protocols on VAP

The meta-analysis shows that nurse-led weaning protocols significantly reduce the incidence of VAP. However, caution is advised because of the low quality of evidence from the included cohort studies. Moreover, it is important to acknowledge that only two studies were available for this outcome, which significantly limits the reliability and generalizability of our findings. The small number of studies per outcome reduces the statistical power of the meta-analysis and increases the possibility that the results are influenced by chance or publication bias. This issue also limits the correct interpretation of the results, and therefore, our conclusions in this regard should be viewed as preliminary until more high-quality research is available.

Our findings are consistent with studies by Dries et al. (30), Marelich et al. (29), and Plani et al. (31), which also reported a reduction in VAP incidence with nurse-led weaning protocols. Conversely, our results differ from studies by Ely et al. (27) and Kollef et al. (28), which did not observe a significant decrease in VAP incidence with nurse-led weaning protocols.

### 4.5 Impact of nurse-led weaning protocols on mortality rate and reintubation rate

Our study indicates that nurse-led weaning protocols do not significantly impact ICU or hospital mortality rates or reintubation rates. These findings align with previous research by Hirzallah et al. (19), Blackwood et al. (32), and Price (36).

### 4.6 Strengths and limitations

To our knowledge, this review is the first comprehensive assessment comparing the effects of nurse-led versus physician-led weaning on mechanically ventilated patients through a systematic review and meta-analysis. This supports the idea that ICU nurses alone can effectively guide liberation from mechanical ventilation. However, several limitations need to be considered in this study. First, it is important to note that the included studies were limited to six countries: South Korea, Thailand, the UK, France, the USA, and the Netherlands. Therefore, caution should be exercised when generalizing these results to other countries or healthcare settings. Second, the number of studies on nurse-led weaning protocols was relatively small, with only six studies identified, including three randomized controlled trials. Thus, interpretations of the findings should be approached with caution due to the limited number and potential variability in study design. Third, a methodological limitation of this review is the inability to blind healthcare personnel to the weaning method due to the nature of nurse-led protocols. Consequently, there is a possibility that the decisions and actions of healthcare personnel were influenced, leading to biased estimates of treatment outcomes. Additionally, the use of different protocols across the six included studies may introduce heterogeneity in the results. Lastly, due to the lack of a meta-analysis involving more than ten studies, publication bias was not assessed in this review. Although efforts were made to identify unpublished studies through grey literature and trial registry searches, no eligible unpublished studies were found.

### 4.7 Implications for future research

Nurse-led weaning protocols have gained significant attention worldwide, with a growing body of evidence suggesting their important role in reducing the duration of MV, LOS in the ICU and hospital, and lowering the incidence of VAP. Our systematic review further confirms the effectiveness of nurse-led weaning protocols in achieving these outcomes. However, it is important to acknowledge the limitations of our study, such as the relatively small number of included articles and the lower quality of some articles. Despite these limitations, given the increasing recognition of nurse-led weaning protocols in global practice, it is essential that research in this area continues and evolves to fully explore their potential.

## 5 Conclusion

Our study findings suggest that nurse-led weaning protocols may have the potential to reduce the duration of MV, LOS in the ICU and hospital, and lower the incidence of VAP in mechanically ventilated patients. However, it is important to interpret these results with caution, as the current evidence base is limited by the small number of included studies and the moderate-to-low quality of evidence for certain outcomes. Moreover, considerable heterogeneity in study design and implementation across the reviewed literature further restricts the generalizability of our conclusions. Therefore, further research, especially large-sample, multicenter randomized controlled trials, is necessary to more definitively evaluate the clinical effectiveness and safety of nurse-led weaning protocols.

## Data Availability

The original contributions presented in the study are included in the article/supplementary material, further inquiries can be directed to the corresponding authors.

## Footnotes

^1^ www.opengrey.eu

^2^ www.greylit.org

## References

[1] Belenguer-Muncharaz A Díaz-Tormo C Granero-Gasamans E Mateu-Campos ML. Protocol-directed weaning versus conventional weaning from mechanical ventilation for neurocritical patients in an intensive care unit: a nonrandomized quasi-experimental study. Crit Care Sci. (2023) 35:44–56. doi: 10.5935/2965-2774.20230340-en, PMID: 37712729 PMC10275310

[2] Marinaki C Kapadochos T Katsoulas T Rubbi I Liveri A Stavropoulou A . Estimation of the optimal time needed for weaning of intensive care unit tracheostomized patients on mechanical ventilation. A prospective observational study. Acta Biomed. (2023) 94:e2023103. doi: 10.23750/abm.v94i2.14243, PMID: 37092617 PMC10210572

[3] Ramaswamy A Kumar R Arul M Ish P Madan M Gupta NK . Prediction of weaning outcome from mechanical ventilation using ultrasound assessment of parasternal intercostal muscle thickness. Indian J Crit Care Med. (2023) 27:704–8. doi: 10.5005/jp-journals-10071-24548, PMID: 37908421 PMC10613859

[4] Marques MR Pereira JM Paiva JA de Casasola-Sánchez GG Tung-Chen Y. Ultrasonography to access diaphragm dysfunction and predict the success of mechanical ventilation weaning in critical care: a narrative review. J Ultrasound Med. (2024) 43:223–36. doi: 10.1002/jum.16363, PMID: 37915259

[5] Park JE Kim DY Park JW Jung YJ Lee KS Park JH . Development of a machine learning model for predicting weaning outcomes based solely on continuous ventilator parameters during spontaneous breathing trials. Bioengineering (Basel). (2023) 10:1163. doi: 10.3390/bioengineering10101163, PMID: 37892893 PMC10604888

[6] Cederwall CJ Plos K Rose L Dübeck A Ringdal M. Critical care nurses' management of prolonged weaning: an interview study. Nurs Crit Care. (2014) 19:236–42. doi: 10.1111/nicc.12092, PMID: 24809683

[7] Pinto-Villalba RS Leon-Rojas JE. Reported adverse events during out-of-hospital mechanical ventilation and ventilatory support in emergency medical services and critical care transport crews: a systematic review. Front Med (Lausanne). (2023) 10:1229053. doi: 10.3389/fmed.2023.1229053, PMID: 37877027 PMC10590890

[8] Pham T Brochard LJ Slutsky AS. Mechanical ventilation: state of the art. Mayo Clin Proc. (2017) 92:1382–400. doi: 10.1016/j.mayocp.2017.05.004, PMID: 28870355

[9] Mancebo J. Weaning from mechanical ventilation. Eur Respir J. (1996) 9:1923–31. doi: 10.1183/09031936.96.09091923, PMID: 8880113

[10] Béduneau G Pham T Schortgen F Piquilloud L Zogheib E Jonas M . Epidemiology of weaning outcome according to a new definition. The WIND study. Am J Respir Crit Care Med. (2017) 195:772–83. doi: 10.1164/rccm.201602-0320OC, PMID: 27626706

[11] Borges LGA Savi A Teixeira C de Oliveira RP De Camillis MLF Wickert R . Mechanical ventilation weaning protocol improves medical adherence and results. J Crit Care. (2017) 41:296–302. doi: 10.1016/j.jcrc.2017.07.014, PMID: 28797619

[12] Gunther I Pradhan D Lubinsky A Urquhart A Thompson JA Reynolds S. Use of a multidisciplinary mechanical ventilation weaning protocol to improve patient outcomes and empower staff in a medical intensive care unit. Dimens Crit Care Nurs. (2021) 40:67–74. doi: 10.1097/DCC.0000000000000462, PMID: 33961373

[13] Matlock DN Perez SM Borchert HA Proffitt BL Peeples SE Chandler AL . Implementing a weaning protocol for noninvasive respiratory support in neonates decreases overuse and length of stay. Respir Care. (2021) 66:644–51. doi: 10.4187/respcare.07985, PMID: 33531357

[14] Foster GH Conway WA Pamulkov N Lester JL Magilligan DJ Jr. Early extubation after coronary artery bypass: brief report. Crit Care Med. (1984) 12:994–6. doi: 10.1097/00003246-198411000-00017, PMID: 6333969

[15] Ward D Fulbrook P. Nursing strategies for effective weaning of the critically ill mechanically ventilated patient. Crit Care Nurs Clin North Am. (2016) 28:499–512. doi: 10.1016/j.cnc.2016.07.008, PMID: 28236395

[16] Chaiwat O Sarima N Niyompanitpattana K Komoltri C Udomphorn Y Kongsayreepong S. Protocol-directed vs. physician-directed weaning from ventilator in intra-abdominal surgical patients. J Med Assoc Thail. (2010) 93:930–6. PMID: 20718169

[17] Danckers M Grosu H Jean R Cruz RB Fidellaga A Han Q . Nurse-driven, protocol-directed weaning from mechanical ventilation improves clinical outcomes and is well accepted by intensive care unit physicians. J Crit Care. (2013) 28:433–41. doi: 10.1016/j.jcrc.2012.10.012, PMID: 23265291

[18] Roh JH Synn A Lim CM Suh HJ Hong SB Huh JW . A weaning protocol administered by critical care nurses for the weaning of patients from mechanical ventilation. J Crit Care. (2012) 27:549–55. doi: 10.1016/j.jcrc.2011.11.008, PMID: 22227086

[19] Hirzallah FM Alkaissi A do Céu Barbieri-Figueiredo M. A systematic review of nurse-led weaning protocol for mechanically ventilated adult patients. Nurs Crit Care. (2019) 24:89–96. doi: 10.1111/nicc.12404, PMID: 30618113

[20] Duyndam A Houmes RJ van Rosmalen J Tibboel D van Dijk M Ista E. Implementation of a nurse-driven ventilation weaning protocol in critically ill children: can it improve patient outcome? Aust Crit Care. (2020) 33:80–8. doi: 10.1016/j.aucc.2019.01.005, PMID: 30876696

[21] Moher D Liberati A Tetzlaff J Altman DG. Preferred reporting items for systematic reviews and meta-analyses: the PRISMA statement. PLoS Med. (2009) 6:e1000097. doi: 10.1371/journal.pmed.1000097, PMID: 19621072 PMC2707599

[22] Sterne JAC Savović J Page MJ Elbers RG Blencowe NS Boutron I . RoB 2: a revised tool for assessing risk of bias in randomised trials. BMJ. (2019) 366:l4898. doi: 10.1136/bmj.l4898, PMID: 31462531

[23] Zhou J Sun Y Ji M Li X Wang Z. Association of Vitamin B Status with risk of dementia in cohort studies: a systematic review and meta-analysis. J Am Med Dir Assoc. (2022) 23:1826.e21–35. doi: 10.1016/j.jamda.2022.05.022, PMID: 35779574

[24] Borenstein M. Research note: in a meta-analysis, the I (2) index does not tell us how much the effect size varies across studies. J Physiother. (2020) 66:135–9. doi: 10.1016/j.jphys.2020.02.011, PMID: 32307309

[25] Tonnelier JM Prat G Le Gal G Gut-Gobert C Renault A Boles JM . Impact of a nurses' protocol-directed weaning procedure on outcomes in patients undergoing mechanical ventilation for longer than 48 hours: a prospective cohort study with a matched historical control group. Crit Care. (2005) 9:R83–9. doi: 10.1186/cc3030, PMID: 15774054 PMC1175918

[26] Rushforth K. A randomised controlled trial of weaning from mechanical ventilation in paediatric intensive care (PIC). Methodological and practical issues. Intensive Crit Care Nurs. (2005) 21:76–86. doi: 10.1016/j.iccn.2004.07.009, PMID: 15778071

[27] Ely EW Baker AM Dunagan DP Burke HL Smith AC Kelly PT . Effect on the duration of mechanical ventilation of identifying patients capable of breathing spontaneously. N Engl J Med. (1996) 335:1864–9. doi: 10.1056/NEJM199612193352502, PMID: 8948561

[28] Kollef MH Shapiro SD Silver P St John RE Prentice D Sauer S . A randomized, controlled trial of protocol-directed versus physician-directed weaning from mechanical ventilation. Crit Care Med. (1997) 25:567–74. doi: 10.1097/00003246-199704000-00004, PMID: 9142019

[29] Marelich GP Murin S Battistella F Inciardi J Vierra T Roby M. Protocol weaning of mechanical ventilation in medical and surgical patients by respiratory care practitioners and nurses: effect on weaning time and incidence of ventilator-associated pneumonia. Chest. (2000) 118:459–67. doi: 10.1378/chest.118.2.459, PMID: 10936141

[30] Dries DJ McGonigal MD Malian MS Bor BJ Sullivan C. Protocol-driven ventilator weaning reduces use of mechanical ventilation, rate of early reintubation, and ventilator-associated pneumonia. J Trauma. (2004) 56:943–52. doi: 10.1097/01.ta.0000124462.61495.45, PMID: 15179231

[31] Plani N Becker P van Aswegen H. The use of a weaning and extubation protocol to facilitate effective weaning and extubation from mechanical ventilation in patients suffering from traumatic injuries: a non-randomized experimental trial comparing a prospective to retrospective cohort. Physiother Theory Pract. (2013) 29:211–21. doi: 10.3109/09593985.2012.718410, PMID: 22943632

[32] Blackwood B Alderdice F Burns KE Cardwell CR Lavery G O'Halloran P. Protocolized versus non-protocolized weaning for reducing the duration of mechanical ventilation in critically ill adult patients. Cochrane Database Syst Rev. (2010) 12:Cd006904. doi: 10.1002/14651858.CD006904.pub2 20464747

[33] Piotto RF Maia LN Machado MN Orrico SP. Effects of the use of mechanical ventilation weaning protocol in the coronary care unit: randomized study. Rev Bras Cir Cardiovasc. (2011) 26:213–21. doi: 10.1590/s0102-76382011000200011, PMID: 21894411

[34] Smyrnios NA Connolly A Wilson MM Curley FJ French CT Heard SO . Effects of a multifaceted, multidisciplinary, hospital-wide quality improvement program on weaning from mechanical ventilation. Crit Care Med. (2002) 30:1224–30. doi: 10.1097/00003246-200206000-00009, PMID: 12072672

[35] Grap MJ Strickland D Tormey L Keane K Lubin S Emerson J . Collaborative practice: development, implementation, and evaluation of a weaning protocol for patients receiving mechanical ventilation. Am J Crit Care. (2003) 12:454–60. doi: 10.4037/ajcc2003.12.5.454, PMID: 14503429

[36] Price AM. Nurse-led weaning from mechanical ventilation: where's the evidence? Intensive Crit Care Nurs. (2001) 17:167–76. doi: 10.1054/iccn.2001.1557, PMID: 11868687

